# Clinical Features, Treatment, and Outcome of HIV-Associated Immune Thrombocytopenia in the HAART Era

**DOI:** 10.1155/2012/910954

**Published:** 2012-05-28

**Authors:** Kimberley L. S. Ambler, Linda M. Vickars, Chantal S. Leger, Lynda M. Foltz, Julio S. G. Montaner, Marianne Harris, Viviane Dias Lima, Heather A. Leitch

**Affiliations:** ^1^Division of Hematology, University of British Columbia, 2775 Laurel Street, Vancouver, BC, Canada V5Z 1M9; ^2^Division of Hematology, St. Paul's Hospital and University of British Columbia, Vancouver, BC, Canada V6Z 1Y6; ^3^BC Centre for Excellence in HIV/AIDS (CFE), University of British Columbia, Vancouver, BC, Canada V6Z 1Y6; ^4^AIDS Research Program, St. Paul's Hospital and the University of British Columbia, Vancouver, BC, Canada V6Z 1Y6

## Abstract

The characteristics of HIV-associated ITP were documented prior to the HAART era, and the optimal treatment beyond HAART is unknown. We performed a review of patients with HIV-associated ITP and at least one platelet count <20 × 10^9^/L since January 1996. Of 5290 patients in the BC Centre for Excellence in HIV/AIDS database, 31 (0.6%) had an ITP diagnosis and platelet count <20 × 10^9^/L. Initial ITP treatment included IVIG, *n* = 12; steroids, *n* = 10; anti-RhD, *n* = 8; HAART, *n* = 3. Sixteen patients achieved response and nine patients achieved complete response according to the International Working Group criteria. Median time to response was 14 days. Platelet response was not significantly associated with treatment received, but complete response was lower in patients with a history of injection drug use. Complications of ITP treatment occurred in two patients and there were four unrelated deaths. At a median followup of 48 months, 22 patients (71%) required secondary ITP treatment. This is to our knowledge the largest series of severe HIV-associated ITP reported in the HAART era. Although most patients achieved a safe platelet count with primary ITP treatment, nearly all required retreatment for ITP recurrence. New approaches to the treatment of severe ITP in this population are needed.

## 1. Introduction

An association between immune thrombocytopenia (ITP) and the acquired immune deficiency syndrome (AIDS) was first recognized in 1982 [1]. Prior to the advent of highly active antiretroviral therapy (HAART), the incidence of HIV-associated thrombocytopenia was estimated at 10–30%, and thrombocytopenia was the initial manifestation of HIV in approximately 10% of cases [2, 3]. The incidence varied according to the definition of thrombocytopenia and the characteristics of the baseline population; for example, it was more common in HIV-infected intravenous drug users (IDUs) compared to HIV-infected men who have sex with men [2]. Although thrombocytopenia may occur at any time during the course of HIV infection, the incidence generally correlates with the degree of immunosuppression and is more prevalent in individuals with clinical AIDS [4–6].

In the initial reports of HIV-associated ITP, thrombocytopenia generally responded well to treatment with corticosteroids or splenectomy [7–9]. Subsequent studies showed improved platelet counts with antiretroviral therapy, in particular HAART [10, 11], and this is now the initial treatment of choice for patients with HIV-associated ITP [12]. Since the era of widespread use of HAART, there have been no published studies describing the incidence or clinical features of HIV-associated ITP [3]. Further, there are minimal data on the safety of various treatment regimens in HIV-positive patients, and the optimal treatment approach to HIV-associated ITP beyond HAART is unknown [3, 13]. The objectives of this study were to determine the frequency of severe HIV-associated ITP in the HAART era and to describe the clinical features, treatment, and outcomes of patients diagnosed with severe HIV-associated ITP in the HAART era.

## 2. Methods

We searched the BC Centre for Excellence in HIV/AIDS (CFE) database to identify patients with HIV who had at least one platelet count <100 × 10^9^/L since January 1996; the year HAART use was widely adopted in British Columbia. For this paper, we chose to focus on patients with severe HIV-associated ITP. Although the International Working Group (IWG) definition of severe ITP includes only patients with clinically relevant bleeding [14], since it would be impossible to identify this group of patients retrospectively from our database, we defined severe thrombocytopenia as at least one platelet count <20 × 10^9^/L. We chose this approach because, at our institution, these patients are routinely referred to hematology and generally require treatment, whereas patients with HIV-associated ITP and higher platelet counts are usually managed by their primary care physician or HIV specialist. We reviewed all charts from patients with a platelet count <20 × 10^9^/L. For the analysis, we included only patients with a diagnosis of HIV-associated ITP made by a hematologist. Patients were excluded if they had pancytopenia, were taking medications that cause thrombocytopenia, had another documented cause of thrombocytopenia, or did not have documentation of assessment by a hematologist. Coinfection with hepatitis B or C was defined as serologic evidence of hepatitis B or C infection prior to or at the time of ITP diagnosis. Chronic liver disease was defined as radiologic or laboratory evidence of chronic liver dysfunction prior to the time of ITP diagnosis. 

The IWG criteria were used to assess response, with complete response (CR) defined as a platelet count ≥100 × 10^9^/L, response (R) defined as a platelet count ≥30 × 10^9^/L and at least a two-fold increase from baseline count and absence of bleeding, no response (NR) defined as failure to achieve a platelet count ≥30 × 10^9^/L or less than a two-fold increase from baseline, or ongoing bleeding, and loss of response defined as recurrent platelet count <30 × 10^9^/L or <two-fold greater than baseline or bleeding [14]. Descriptive statistics were used to summarize the data. Response rates were compared using the Fischer's exact test, using SPSS for Windows, version 18.0.

## 3. Results

**Figure 1:**
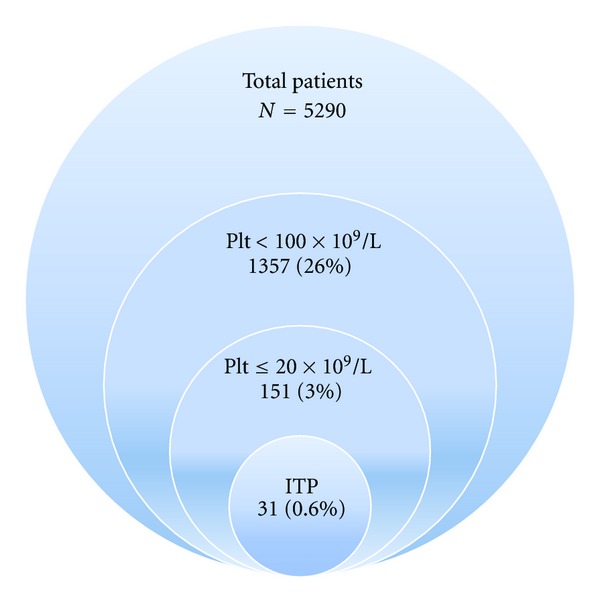
Incidence of thrombocytopenia in HIV-infected individuals in the HAART era.

Of 5290 patients in the CFE database since 1996, 1357 (26%) had at least one platelet count <100 × 10^9^/L, 417 (8%) had at least one platelet count <50 × 10^9^/L, 151 (3%) had at least one platelet count ≤20 × 10^9^/L, and 31 patients (0.6%) were diagnosed with HIV-associated ITP and had a platelet count ≤20 × 10^9^/L, see Figure 1.

### 3.1. Clinical Characteristics

The clinical characteristics of the 31 patients diagnosed with severe HIV-associated ITP are shown in Table 1. The median age at ITP diagnosis was 37 (range 27–66) years. The median platelet count was 10 × 10^9^/L (range 2–19 × 10^9^/L), median hemoglobin was 129 (34–165) g/L, and median neutrophil count was 2.5 (1.0–17.0) × 10^9^/L. Seventeen patients (55%) presented with clinical bleeding and four (13%) required packed red blood cell transfusion. Comorbidities included chronic liver disease in five patients, chronic renal failure in two, seizure disorder in two, and chronic obstructive pulmonary disease, Crohn's disease, type 2 diabetes mellitus, peripheral vascular disease, phenylketonuria, prostate cancer, psychiatric disorder, and pulmonary hypertension each in one patient. Twelve patients had hepatitis C and four patients had hepatitis B coinfection. Treatment for the coinfections was not documented. Six patients had a bone marrow aspirate and biopsy and all showed increased megakaryocyte numbers, consistent with a diagnosis of ITP. Of 29 patients with a CD4 count documented at the time of severe ITP diagnosis, the median CD4 was 290 (20–600)  cells/*μ*L. Four patients had an HIV viral load documented at the time of severe ITP diagnosis, and it was >100 000 copies/mL in two patients, 92730 and 90 copies/mL in each of the other patients. Ten patients were receiving HAART at the time of severe ITP diagnosis. Of these, three had a CD4 < 200  cells/*μ*L, suggesting suboptimal therapy or adherence to therapy.

### 3.2. Primary ITP Treatment

Treatments received at the first episode of a platelet count <20 × 10^9^/L are listed in Table 2. Thirteen patients received HAART as part of their primary ITP treatment, including the ten patients who were receiving HAART prior to the diagnosis of ITP. Of the 18 patients who did not receive HAART with primary ITP therapy, 12 had a CD4 count >200  cells/*μ*L and 13 had IDU as their risk factor for HIV, including the six patients with a CD4 count <200  cells/*μ*L. 

**Figure 2:**
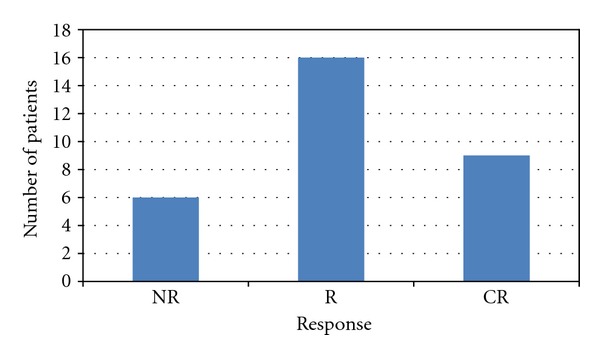
Maximum platelet response.

Overall, 25 patients achieved a CR or R according to the IWG criteria (see Figure 2). The median platelet response within 30 days was 58 (5–322) × 10^9^/L and the median time to R was 14 (1–3192) days.

### 3.3. Secondary ITP Treatment

At a median followup of 48 (0.2–138.5) months, 22 patients (71%) had a loss of response and required secondary ITP treatment for a recurrent platelet count <20 × 10^9^/L. The median platelet count at recurrent ITP diagnosis was 10 (5–20) × 10^9^/L. The median time to loss of response was 39 (8–858) days. Of 13 patients that received HAART with their primary treatment, seven had recurrent ITP. Six of seven patients who did not relapse received HAART with their primary treatment, three received HAART as treatment for ITP, and three were on HAART prior to ITP diagnosis. 

 Secondary treatment included IVIG in nine patients, anti-RhD in six patients, steroids in four patients, splenectomy in three patients, and initiation of HAART in one patient. The median platelet response within 30 days of the initiation of secondary ITP treatment was 42 (21–198) × 10^9^/L (*n* = 19). Eleven patients achieved R and eight patients achieved CR to secondary treatment. The median time to response was seven (2–280) days. All three patients who underwent splenectomy achieved a normal platelet count which was sustained at followup of 14, 58, and 123 months.

### 3.4. Predictors of ITP Response

The probability of response to primary ITP treatment did not appear to be significantly influenced by the choice of treatment or by the number of treatments received; however, firm conclusions regarding this are limited by small patient numbers. When considering patient characteristics, patients with a history of IDU were less likely to achieve a CR (*P* = 0.04, *N* = 30). We did not find a significant difference in any patient characteristics between patients who achieved R compared to those who did not, likely because the number of patients with NR was so small. Loss of response did not appear to be associated with any particular patient or treatment characteristic.

### 3.5. Complications of Treatment

Complications of treatment were psychiatric effects of steroids in one patient and postsplenectomy fever and hematoma in one patient. There were no opportunistic infections; however, seven patients received prophylaxis for *Pneumocystis jirovecii* pneumonia (PCP), including five patients who received septra, and two patients who received dapsone.

### 3.6. Survival and Causes of Death

There were four deaths and causes were variceal bleed in two patients, Evan's syndrome and hepatic failure in one patient, and advanced HIV in one patient. Both patients who died of variceal bleeding had a platelet count >50 × 10^9^/L at most recent followup, a history of IDU, hepatitis C coinfection, and hepatic cirrhosis. The patient who died of Evan's syndrome had sexual contact as his risk factor for HIV and no history of hepatitis C or chronic liver disease. He was receiving HAART prior to death and the most recent CD4 was 260  cells/*μ*L. His platelet count on the day of death was 8 × 10^9^/L. The patient who died of advanced HIV had a history of coinfection with hepatitis C, PCP, TB, and pulmonary hypertension. The most recent platelet count was 23 × 10^9^/L nine days before her death.

## 4. Discussion

In this study, although the incidence of thrombocytopenia in HIV-infected individuals was high (26%), the incidence of severe HIV-associated thrombocytopenia was only 0.6%. These results are similar to those of studies performed in the era prior to the widespread use of HAART, in which the incidence of platelet count less than 150 × 10^9^/L was 10–30% in HIV-infected individuals, and the incidence of a platelet count less than 50 × 10^9^/L was 1.5–9% [2, 4, 15]. Approximately two-thirds of patients in our study had a CD4 count over 200 cells/*μ*L. Although this seems contrary to previous studies that showed an increased incidence and severity of thrombocytopenia with lower CD4 counts [4–6], in our study, the higher number of patients with CD4 count greater than 200 cells/*μ*L is most likely a reflection of the composition of the baseline population in the HAART era. Importantly, our study shows that control of HIV infection alone is not sufficient to prevent ITP in all patients.

 In our population in the HAART era, more patients with severe thrombocytopenia had a history of IDU than sexual contact as their HIV risk factor, consistent with findings in the pre-HAART era [2, 8, 15]. We also found that patients with a history of IDU had inferior platelet response. Potential reasons for this association include higher rates of coinfection with hepatitis C in the IDU population, lower rates of compliance with HAART and other treatments, direct toxicity from the injection drugs, or from contaminating substances mixed in the drugs. In our study the type of drugs and the potential for contaminating substances were not documented, but we did find a high incidence of coinfection with hepatitis C virus, which is consistent with the findings from prior studies [2, 16]. 

Nearly all patients in this study achieved a safe platelet count with primary ITP treatment. The rates of response to steroids, IVIG, and anti-D were similar to those previously reported and we were unable to show a significant difference in response according to treatment received. In a study of injection drug users with HIV-associated ITP in the pre-HAART era, Landonio showed response rates of 65% and 80% to IVIG and anti-D [8]. Walsh found that 16 of 17 men who have sex with men with ITP in the pre-HAART era responded to prednisone [7].

Although there were few treatment complications in our study, most patients (71%) required secondary treatment for loss of response, including seven of 13 patients receiving HAART with initial ITP therapy. This is consistent with findings from the study by Landonio, in which only four of 17 patients treated with IVIG had sustained remissions, and all patients treated with steroids had recurrent ITP after discontinuation of initial therapy [8]. In a randomized cross-over study of nine patients with severe HIV-associated ITP, Scaradavou found the median duration of response was 19 days following treatment with IVIG and 41 days following anti-RhD [17].

As with primary treatment for ITP, most patients in our study who received secondary treatment for recurrent thrombocytopenia achieved a platelet count >30 × 10^9^/L. Although splenectomy appeared to be safe and effective in the three patients who underwent this treatment, given the lifelong increased risk of severe infections, we have reservations about using this therapy in patients with HIV, particularly those with hepatitis C coinfection. In a series of 68 patients who underwent splenectomy for HIV-associated ITP in the pre-HAART era, 82% had sustained platelet counts >50 × 10^9^/L and there was no significant difference in progression to AIDS or survival compared to patients without splenectomy. However, two patients died of fulminant septic shock and two others presented with *Streptococcus pneumoniae* meningitis [18].

This is to our knowledge the largest series of patients with severe HIV-related ITP reported since the availability of HAART, and one strength of the study is the substantial length of followup, a median of four years. The main limitation of our study is its retrospective nature. Because of this, small patient numbers, and many variables in this patient population, we cannot make definitive conclusions about the efficacy of one treatment compared to another. In addition, since the study was conducted at a single institution, the results may not apply to other populations. Furthermore, since we chose only to include patients with severe HIV-associated ITP who were referred to hematology, there is selection bias and the findings are not necessarily applicable to patients with mild or moderate thrombocytopenia. Finally, none of the patients in our study were treated with rituximab or thrombopoietin receptor agonists; therefore, we cannot comment on the safety or efficacy of these newer agents in the treatment of HIV-associated ITP.

## 5. Conclusions

HIV-associated ITP remains an important clinical problem in the era of the widespread use of HAART. Although most patients respond to primary ITP treatment and there are few treatment-related complications, nearly all patients require retreatment for recurrent ITP. Inferior response to treatment was associated with a history of IDU. New approaches to the treatment of HIV-associated ITP in this patient population are needed.

##  Conflict of Interests

The authors have no relevant conflict of interests to disclose.

## Figures and Tables

**Table 1:** Clinical characteristics of 31 patients with HIV-associated ITP diagnosed since 1996.

Characteristic	*N*
Age at ITP presentation (years)	
≤40	12
>40	19
Gender	
Male	25
Female	5
Transgender (MF)	1
Platelet count (×10^9^/L)	
≤10	15
>10	16
Hemoglobin at ITP diagnosis (g/L)	
<90	4
>90	24
Clinical bleeding	17
Site of bleeding	
Epistaxis	14
Menorrhagia	3
Gingival bleed	2
Gastrointestinal bleed	1
Hemoptysis	1
HIV risk factor (*N* = 26)	
Sexual	10
IDU	16
CD4 at ITP diagnosis (cells/*μ*L, *N* = 29)	
<200	8
≥200	21
Prior AIDS^1^	5
Coinfections	
Hepatitis B	4
Hepatitis C	12
Receiving HAART^2^ at ITP diagnosis	
No	20
Yes	10
Comorbidities	15

*¹*Mycobacterium avian complex, *n* = 2; *Pneumocystis jirovecii* pneumonia,  *n* = 2; anal condylomata, *n* = 1.

^2^HAART was 1 nucleoside analog (NA), 1 protease inhibitor, and either a 2nd NA or a nonnucleoside reverse transcription inhibitor.

**Table 2:** Treatment regimens for HIV-associated ITP and responses achieved.

Treatment	Dose	*N*	Bleeding (%)	R + CR (%)	CR (%)	Median time to R in days (range)	Relapse (%)
IVIG	1 g/kg/day × 2 days	7	6 (86)	5 (71)	4 (57)	4 (3–9)	6 (86)
							
Anti-D	2.4–4 mg	7	2 (29)	6 (86)	0 (0)	14 (1–61)	4 (57)
							
Prednisone	50–85 mg daily	4	2 (50)	4 (100)	1 (25)	4.5 (3–13)	3 (75)
							
HAART alone	*See footnote	4	1 (25)	3 (75)	1 (25)	267 (1–1379)	3 (75)
							
IVIG +	1 g/kg/day × 2 days	5	4 (80)	5 (100)	3 (60)	11 (3–16)	3 (60)
Prednisone	50–70 mg						
							
Anti-D +	1.3 mg	1	1 (100)	1 (100)	0 (0)	22	1 (100)
Prednisone	40 mg daily						
							
HAART +	**See footnote	9	5 (56)	8 (89)	5 (56)	13.5 (3–22)	4 (44)
Other therapy							
							
None		3	0 (0)	2 (67)	2 (67)	696 (26–3192)	2 (67)

*HAART was 1 nucleoside analog (NA), 1 protease inhibitor, and either a 2nd NA or a nonnucleoside reverse transcription inhibitor.

**Other therapy was IVIG, prednisone, or anti-D in the doses listed above. Patients in this group were also included in the groups above with patients who received the respective therapies without HAART.
